# Chlorhexidine decontamination of sputum for culturing *Mycobacterium tuberculosis*

**DOI:** 10.1186/s12866-015-0479-4

**Published:** 2015-08-05

**Authors:** Shady Asmar, Michel Drancourt

**Affiliations:** Aix Marseille Université, URMITE, UM63, CNRS 7278, IRD 198, Inserm 1095, Institut Hospitalo-Universitaire «Méditerranée Infection», AP-HM, 13005 Marseille, France; Unité de Recherche sur les Maladies Infectieuses et Tropicales Emergentes, Faculté de Médecine, 27, Boulevard Jean Moulin, 13385 Marseille, Cedex 5 France

**Keywords:** *Mycobacterium tuberculosis*, Decontamination, Chlorhexidine, Squalamine, N-acetyl-cysteine-sodium chloride, Culture, Contamination, Sensitivity, Standards for Reporting of Diagnostic Accuracy (STARD)

## Abstract

**Background:**

Culture of *Mycobacterium tuberculosis* is the gold standard method for the laboratory diagnosis of pulmonary tuberculosis, after effective decontamination.

**Results:**

We evaluated squalamine and chlorhexidine to decontaminate sputum specimens for the culture of mycobacteria. Eight sputum specimens were artificially infected with 10^5^ colony-forming units (cfu)/mL *Mycobacterium tuberculosis* and *Staphylococcus aureus, Pseudomonas aeruginosa* and *Candida albicans* as contaminants. In the second step, we tested chlorhexidine-based decontamination on 191 clinical specimens, (Chlorhexidine, 0.1, 0.5 and 0.7 %). In a last step, growth of contaminants and mycobacteria was measured in 75 consecutive sputum specimens using the routine NALC-NaOH decontamination protocol or with 0.7 % chlorhexidine decontamination and an inoculation on Coletsos medium.

In the artificially model, contaminants grew in 100 % of the artificially infected sputum specimens decontaminated using 100 mg/mL squalamine, in 62.5 % of specimens decontaminated using N-Acetyl-L-Cysteine-Sodium Hydroxide (NALC-NaOH), and in 0 % of specimens decontaminated using 0.1 %, 0.35 %, or 1 % chlorhexidine (*P* < 0.05). These specimens yielded <10^2^ cfu *M. tuberculosis* using NALC-NaOH and > 1.4.10^2^ cfu *M. tuberculosis* when any concentration of chlorhexidine was used (*P* < 0.05).

In the second step we found that 0.7 %-chlorhexidine yielded 0 % contamination rate, 3.2 % for 0.5 %-chlorhexidine and 28.3 % for 0.1 %-chlorhexidine. As for the 75 specimens treated in parallel by both methods we found that when using the standard NALC-NaOH decontamination method, 8/75 (10.7 %) specimens yielded *M. tuberculosis* colonies with a time to detection of 17.5 ± 3 days and an 8 % contamination rate. Additionally, 14 specimens yielded mycobacteria colonies (12 *M. tuberculosis*, and 2 *Mycobacterium bolletii*) (18.7 %) (*P* = 0.25), which has yielded a 100 % sensitivity for the chlorhexidine protocol. Time to detection was of 15.86 ± 4.7 days (*P* = 0.39) and a 0 % contamination rate (*P* < 0.05) using the 0.7 %-chlorhexidine protocol.

**Conclusion:**

In our work we showed for the first time that chlorhexidine based decontamination is superior to the standard NALC-NaOH method in the isolation of *M. tuberculosis* from sputum specimens. We currently use 0.7 %-chlorhexidine for the routine decontamination of sputum specimens for the isolation of *M. tuberculosis* and non-tuberculosis mycobacteria on egg-lecithin containing media.

## Background

The isolation and culture of *Mycobacterium tuberculosis* is the gold standard for laboratory pulmonary tuberculosis diagnoses, with a yet unsurpassed sensitivity of 100 colony-forming units per milliliter (cfu/mL) [1,2]*. M. tuberculosis* can be cultured from respiratory tract and stool specimens for pulmonary tuberculosis diagnoses [3,4]. In such specimens, the overgrowth of contaminants, including *Staphylococcus aureus*, *Pseudomonas aeruginosa* and *Candida albicans*, hampers the efficient isolation and culture of *M. tuberculosis* [5]. Various decontamination protocols have been proposed to limit the overgrowth of contaminants, in addition to adding antibiotics and antifungics to the culture media [6]. All of these protocols were shown to inhibit the viability of *M. tuberculosis* to some extent [7]. Sodium chloride (NaOH) -based protocols including the Petroff’s method and the NALC-NaOH method, are widely used for decontamination [8,9], despite the observation that NaOH can also affect *M. tuberculosis* viability; it was shown to kill up to 60 % of *M. tuberculosis* bacilli in clinical specimens [7]. This may yield false negative results, especially in paucibacillary infection cases, such as those observed in HIV-infected patients [10].

Limited data have indicated that a chlorhexidine-based decontamination protocol, once proposed by our laboratory [11] and further used to decontaminate sputum [12,13] and stool specimens [4], performed better than some other methods for sputum specimen decontamination [11]. However, these studies were limited to the recovery of non-tuberculosis mycobacteria. Chlorhexidine has not been thoroughly compared to the reference NaOH protocol for the laboratory diagnosis of pulmonary tuberculosis. Additionally, we recently observed that the broad-spectrum, water-soluble cationic amino-sterol squalamine 1 (7,24-dihydroxylated-24 sulfated cholestane conjugated to spermidine group at C-3) [14] was ineffective against *M. tuberculosis* killing, with a minimum inhibitory concentration (MIC) >100 mg/L; therefore, it is a promising decontaminant [15]. Indeed, squalamine was revealed to have a high efficiency against gram-positive and gram-negative bacteria, with particular efficacy against *S. aureus* (MIC, 3.12 mg/L) and *P. aeruginosa* (MIC, 8 mg/L) as well as some activity against *C. albicans* (MIC >20 mg/L mg/L) [16]. Therefore, squalamine has the potential to be incorporated into decontamination protocols for the isolation of *M. tuberculosis*.

Here, we assessed various decontamination protocols by combining various concentrations of chlorhexidine combined with squalamine for the efficient culture of *M. tuberculosis* from sputum.

## Methods

### Artificially infected sputum specimens

Clinical isolates of *C. albicans*, *P. aeruginosa* and *S. aureus* were calibrated to a final concentration of 10^7^ cfu/mL. Four *M. tuberculosis* strains (H37Rv CIP 104475 and three clinical isolates) were cultured on Coletsos (bioMérieux, Craponne, France), and the colonies were suspended in Mycobacteria Growth Indicator Tubes [(MGIT) Becton Dickinson, Le Pont-de-Claix, France]. The suspensions were rigorously vortexed using 3-mm sterile glass beads (Sigma-Aldrich, Saint-Quentin-Fallavier, France) and bypassed four times through a 25-G needle in order to disperse any clumped cells. The homogenized suspensions were then calibrated to 10^7^ cfu/mL by optical density at 580 nm (Cell Density Meter; Fisher Scientific, Illkirch, France). Smear-negative sputum specimens that were prospectively collected from our laboratory were mixed, sterilized by autoclaving at 121 °C for 15 min and centrifuged at 2500 g for 5 min. The supernatants were inoculated with *P. aeruginosa*, *C. albicans* and *S. aureus* in addition to one of the four different *M. tuberculosis* suspensions in order to achieve a final concentration of 10^5^ cfu/mL for each microorganism.

### The decontamination procedures

For the chlorhexidine or squalamine decontamination protocols, an equal volume of 0.1 % dithiothreitol (Sigma-Aldrich) and sputum specimens were mixed together for 10 min in a 50-mL conical tube. Then, a triple volume of chlorhexidine (0.1, 0.35 or 1 %) (Chlorhexidine digluconate, Sigma-Aldrich) or squalamine solution were added to yield a 100 mg/L final concentration. The samples were vortexed and incubated for 15 min at room temperature with a continuous agitation. In the cases when inoculations were conducted on 5 % sheep-blood Columbia agar (COS, bioMérieux), 10 mL of neutralizing solution [200 mL phosphate buffered saline (PBS), 0.6 g egg-lecithin and 2 mL Tween 80] was added to the chlorhexidine-decontaminated specimens in order to inactivate the chlorhexidine, and the mix was vortexed and incubated for 5 min at room temperature. Next, PBS was added up to 40-mL, the suspension was centrifuged at 1700 g for 15 min, the supernatant was discarded, and the pellet was resuspended in 0.5 mL of sterile PBS, pH 6.8. For the squalamine-chlorhexidine protocol, the squalamine was added first and mixed followed by incubation for 10 min at room temperature, and the chlorhexidine was then added. The reference N-Acetyl-L-Cysteine-Sodium Hydroxide (NALC-NaOH) method was applied as previously described [9].

A 100-μL volume of specimen decontaminated by chlorhexidine at different concentrations (0.1, 0.35 and 1 %), squalamine-100 μg/mL or chlorhexidine-0.1 %-squalamine-100 μg/mL and NALC-NaOH and artificially infected specimens was inoculated in parallel in duplicate on COS with a non-inoculated negative control to measure the survival of the contaminants and in triplicate on a modified Middlebrook 7H10 medium (MOD4), which was previously described [17], with a non-inoculated negative control to compare the *M. tuberculosis* viability using these different protocols. For the contaminants, colonies were observed after 24- and 48-h incubation at 37 °C under a 5 %-CO_2_ atmosphere, into an incubator. Contaminant identification was confirmed using matrix-assisted laser desorption ionization-time of flight mass spectrometry (MALDI-TOF-MS), as previously described [18]. For the *M. tuberculosis*, microcolonies were detected on MOD4 by autofluorescence, as previously described [17].For each Petri-dish of the inoculated media, five random autofluorescence photos were taken on the fifth day of incubation in microaerophilic atmosphere at 37 °C in order to determine the mean number of microcolonies that developed per microscopic field (x 12.5 magnification).

### Chlorhexidine decontamination of clinical sputum specimens

The samples used in this study, were sent to the Mycobacteria Reference Laboratory (Institut Méditerranée Infection, Marseille, France) from January to March 2014. This study has received the agreement of the Ethics Committee of this Institut on February 19, 2007.

A total of 191 sputum specimens including six Ziehl-positive specimens collected in 97 patients suspected of pulmonary tuberculosis, were prospectively decontaminated by chlorhexidine to measure the contamination ratio and recovery of mycobacteria. Based on on-going results, a total of 53 specimens were decontaminated using 0.1 %-chlorhexidine, 55 specimens using 0.5 %-chlorhexidine, and 83 specimens using 0.7 %-chlorhexidine.

Furthermore, 75 other sputum specimens were evaluated for decontamination and recovery of mycobacteria by comparing the routine NALC-NaOH decontamination protocol and a 0.7 %-chlorhexidine decontamination. A 250 μL-volume of decontaminated specimen was inoculated in a Coletsos tube. The colonies were counted and identified with Ziehl-Neelsen staining and quantitative real-time polymerase chain reaction (qPCR), which targeted the internal transcribed spacer (ITS) by incorporation of a forward (5'-GGTGGGGTGTGGTGTTTGAG-3') and a reverse primer (5'-CACGTCCTTCATCGGCTCTC-3') as well as a *M. tuberculosis* probe (6FAM- GCTAGCCGGCAGCGTATCCAT). In the cases when the qPCR failed, colony identification was accomplished by standard 16S rRNA gene PCR sequencing, as previously described [19]. All these experimental procedures were done by one of us (SA) who has particular expertise in the field.

### Statistical analyses

The Fisher exact test was used to assess the significance of the differences in the contamination and isolation rates. The Kruskal-Wallis test was used to assess any significant differences in the microcolony numbers.

## Results

### The artificially infected sputum specimens

Using the reference NALC-NaOH protocol, we observed <7 colonies for any of the contaminants in 5/8 (62.5 %) inoculated COS medium plates. These contaminants were identified as *C. albicans* and *P. aeruginosa* by MALDI-TOF-MS. Using squalamine, we observed an uncountable number of colonies, which were identified as *S. aureus, P. aeruginosa* and *C. albicans* in 8/8 (100 %) specimens (*P* < 0.05). Using 1 %, 0.35 %, and 0.1 % chlorhexidine and 0.1 % - chlorhexidine/100 mg/L - squalamine, we observed no growing contaminants (0 %) (*P* = 0.026, Fisher exact test). For the four *M. tuberculosis* strains, the mean microcolony number that was detected per field resulting from the different decontamination methods are presented in Table 1. There was a significant difference in the *M. tuberculosis* colony number between the different chlorhexidine-based protocols and the reference NALC-NaOH method (*P* < 0.05). The difference in the *M. tuberculosis* colony number between the 0.1 %-chlorhexidine treatment and the other chlorhexidine concentrations was significant (*P* < 0.05). The difference between the 0.35 %-chlorhexidine and 1 %-chlorhexidine treatments as well as between the 0.1 %-chlorhexidine and 0.1 %-chlorhexidine/100 μg/ml-squalamine treatments was not statistically significant (*P* > 0.05).

**Table 1:** Mean, median and standard deviation in the number of *M. tuberculosis* microcolonies, after decontamination of artificially infected sputum samples

*M. tuberculosis* H37Rv CIP 104475	Mean	Median	Standard variation
chlorhexidine −0.1 %	844.4	736.5	361
chlorhexidine −0.1 % - squalamine 100 μg/mL	1288	1396	576.3
chlorhexidine −0.35 %	477.1	453	154.5
chlorhexidine −1 %	665	630	207.6
NALC-NaOH	71.9	66	58.8
*M. tuberculosis* clinical isolate 1	Mean	Median	Standard variation
chlorhexidine-0.1 %	2493.1	2624.5	553.2
chlorhexidine-0.1 % - squalamine 100 μg/mL	1774.5	1309	498.2
chlorhexidine −0.35 %	1259.6	1238.5	393.1
chlorhexidine −1 %	1426.6	1434	357.5
NALC-NaOH	81.7	73	37.3
*M. tuberculosis* clinical isolate 2	Mean	Median	Standard variation
chlorhexidine-0.1 %	556.1	621	155.9
chlorhexidine-0.1 % - squalamine 100 μg/mL	440.4	433	134.6
chlorhexidine −0.35 %	237.9	202	127.4
chlorhexidine −1 %	184.7	180	50.7
NALC-NaOH	4.4	4	2.83
*M. tuberculosis* clinical isolate 3	Mean	Median	Standard variation
chlorhexidine-0.1 %	288.1	284.5	87.8
chlorhexidine-0.1 % - squalamine 100 μg/mL	124.4	136	26.1
chlorhexidine −0.35 %	174.9	194	76.5
chlorhexidine −1 %	140.7	142.5	25.5
NALC-NaOH	4.9	3	3.7

### The clinical specimens

The rate of contamination was respectively 28.3 % (15/53), 3.2 % (2/55) and 0 % for 0.1 %-chlorhexidine, 0.5 %-chlorhexidine (*P* < 0.05) and 0.7 %-chlorhexidine (*P* < 0.05), respectively. Contaminants were identified as *S. aureus* (2) and *Streptococcus* spp. (13). Specimens decontaminated by 0.1 %-chlorhexidine yielded three *M. tuberculosis* isolates, with a mean time to detection of 14.67 ± 5.1 days (9–19). Nine mycobacteria were cultured from specimens treated by 0.7 %-chlorhexidine, including 7 M. *tuberculosis* isolates and 2 *M. avium* isolates, with a 23 ± 4.6 days (17–30) mean time to detection (*P* < 0.05).

**Fig. 1:**
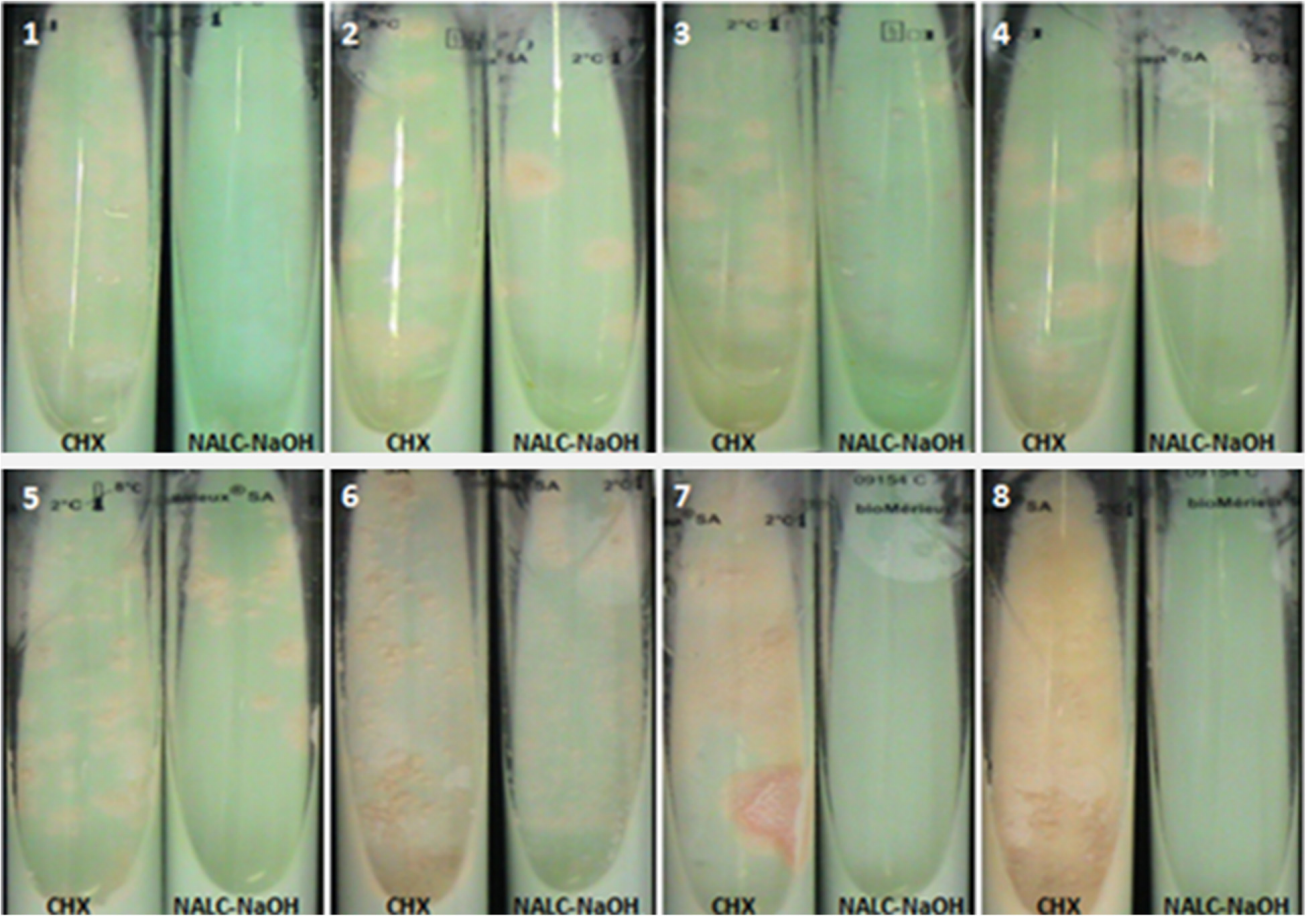
An illustration of the number of mycobacteria colonies developed on Coletsos medium after decontamination by 0.7 %-chlorhexidine or NALC-NaOH (1, 2, 3, 4, 5 and 6 represent *M. tuberculosis* isolates, 7 and 8 represent *M. bolletii* isolates)

As for the 75 sputum specimens treated in parallel by the reference method and 0.7 %-chlorhexidine, NALC-NaOH decontamination yielded eight (10.7 %) *M. tuberculosis* isolates, which grew on Coletsos medium, with a mean time to detection of 17.5 ± 3 days (15–23 days) and an 8 % contamination rate. The contaminants were identified as *Candida orthopsilosis* (1), *Candida glabrata* (1), *Streptococcus oralis* (2), *Hafnia alvei* (1) and *Raoultella ornithinolytica* (1). Using a 0.7 %-chlorhexidine decontamination treatment, 14 mycobacteria (12 *M. tuberculosis* and 2 *Mycobacterium bolletii*) (18.7 %) isolates were observed (*P* = 0.25), with a 100 % sensitivity for *Mycobacterium* isolation when the NALC-NaOH is considered as the reference method with a mean time to detection of 15.85 ± 4.7 days (8–25 days) (*P* = 0.39) for all mycobacteria and 17.33 ± 3.08 days (14–25 days) for the *M. tuberculosis* colonies (*P* = 0.9) and 0 % contamination (*P* < 0.05). All of the *M. tuberculosis* isolates that were observed using the NALC-NaOH decontamination method were also isolated with 0.7 %-chlorhexidine (Table 2). The number of *M. tuberculosis* colonies that were cultured using the 0.7 %-chlorhexidine decontamination method (8, 47, 52, 83, >100, >100, >100 and >100 colonies) was higher than those grown after the NALC-NaOH treatment (12, 7, 8, 31, 13, 17, 66 and >100 colonies) (Table 3, Fig. 1).

**Table 2:** Yield of mycobacteria and contaminants in 75 sputum samples, decontaminated by 0.7 %-chlorhexidine or NALC-NaOH

		NALC-NaOH					
		Positive *M. tuberculosis*	Positive other mycobacteria	Contaminated/Positive	Contaminated/Negative	Negative	Total
Chlorhexidine-0.7 %	Positive *M. tuberculosis*	7	0	1	2	2	12
	Positive other mycobacteria	0	0	0	0	2	2
	Contaminated/Positive	0	0	0	0	0	0
	Contaminated/Negative	0	0	0	0	0	0
	Negative	0	0	0	3	58	61
	Total	7	0	1	5	62	75

**Table 3:** Number of *M. tuberculosis* colonies for the eight positive cultures on Coletsos medium in both 0.7 % chlorhexidine and NALC-NaOH decontamination

	Number of *M. tuberculosis* colonies detected	
Specimen	0.7 % Chlorhexidine	NALC-NaOH
1	8	12
2	47	7
3	52	8
4	83	31
5	>100	13
6	>100	17 (+contamination)
7	>100	66
8	>100	>100

## Discussion

The routine NALC-NaOH decontamination method for sputum specimens does not prevent all contaminations, as a 2–5 % ratio of contamination is routinely observed [20], some works reported higher contamination rate on LJ (10–14 %) [21–23]. Here, we used artificially contaminated sputum in a first step to assess the efficacy of decontamination. Accordingly, a 8 % contamination rate may be due to the fact that we incorporated several specimens from the same cystic fibrosis patients (13.3 %) which are usually contaminated with resistant flora [24]. Additionally, this protocol is known to alter the viability of *M. tuberculosis* in decontaminated sputum, as observed in this study [12]. We therefore compared alternative decontamination protocols.

Based on its reported activity against contaminants [16] and its lack of activity against mycobacteria, including *M. tuberculosis* [15], squalamine was a potential candidate for decontamination. However, our investigation evaluating artificially infected sputum specimens indicated that 100 mg/L squalamine did not eliminate contaminant bacteria. This observation contrasts with the observation that only *C. albicans* resists to 100 mg/mL squalamine (not published data). This agree with a previously published report that showed a MIC of chlorhexidine of >20 mg/L [16]. These data suggest that sputum may contain interfering factors that further limit the activity of squalamine against *P. aeruginosa* and *S. aureus*.

Chlorhexidine was more effective than the routine NALC-NaOH protocol in decontaminating artificially infected and clinical sputum specimens at a 0.1, 0.35 and 1 % concentration in replicate. Moreover, it yielded 10–100 more *M. tuberculosis* colonies than the standard NALC-NaOH method in triplicates, in agreement with previously published data regarding growth of non-tuberculosis mycobacteria on Löwenstein-Jensen medium [12,13].

Contrarily to the results obtained previously, we observed that a 0.1 %-chlorhexidine concentration and a 100 mg/mL squalamine are ineffective for the decontamination of sputum (28.3 % contamination rate). Therefore, we increased the concentration of chlorhexidine and found that a 0.7 %-chlorhexidine was optimal for a routine use.

We therefore used an optimized 0.7 % chlorhexidine decontamination method for clinical sputum specimens and observed that this protocol yielded a non-significant, nevertheless, higher mycobacteria isolate number than the routine NALC-NaOH decontamination method (14 versus 8 isolates in 75 sputum specimens). Moreover, a higher *M. tuberculosis* colony number was also observed, which is in agreement with the results obtained in the artificial model. These observations extended to *M. tuberculosis* and any sputum specimen, which is in line with observations previously made for non-tuberculosis mycobacteria isolated from cystic fibrosis sputum [12,13]. It must be emphasized that chlorhexidine, which is potentially toxic for mycobacteria, must be inactivated by incorporation with egg-lecithin in the culture medium.

## Conclusions

Here, we observed that a chlorhexidine-based decontamination method is effective against contaminants and more appropriate for the viability of mycobacteria. Moreover, we observed for the first time that routine chlorhexidine-based decontamination on specimens including those received from cystic fibrosis patients yielded superior results for the isolation, in particularly of *M. tuberculosis* than the standard NALC-NaOH decontamination method. At last, our preliminary cost evaluation based on 1000 specimens, indicates a 0.31 €/specimen cost of chlorhexidine lower than to the 1.4 €/specimen cost of reference NALC-NaOH decontamination. These tabulations do not take into consideration the short 24-h expire delay for NALC-NaOH; when chlorhexidine can be stored for 3 months at room temperature.

We currently use a 0.7 % chlorhexidine decontamination protocol combined with cultures on an egg-based medium [17] in our routine practice.

## Abbreviations

CFU: Colony forming unit
NALC-NaOH: N-acetyl-L-cystein-sodium chloride
HIV: Human immunodeficiency virus
MIC: Minimum inhibitory concentration
MGIT: Mycobacteria growth indicator tube
MALDI-TOF-MS: Matrix-assisted laser desorption ionization-time of flight mass spectrometry
qPCR: Quantitative real-time polymerase chain reaction
ITS: Internal transcribed spacer
COS: Colombia agar + 5 % sheep blood
